# High-dose buprenorphine inductions in hospital settings

**DOI:** 10.1016/j.ajmo.2025.100118

**Published:** 2025-09-16

**Authors:** Scott A. Wu, Gayane N. Archer, Brent D. Schnipke

**Affiliations:** a Northwestern University Feinberg School of Medicine, Chicago, Ill; b Northwestern Medicine Department of Psychiatry and Behavioral Sciences, Chicago, Ill

**Keywords:** Buprenorphine, High-dose buprenorphine induction, Opioid use disorder, Precipitated withdrawal, Substance use disorder

## Abstract

• We present a discussion of inpatient high-dose buprenorphine induction (HDBI) with theoretical examples.
• Previous reports focus mainly on HDBI performed in emergency departments.
• HDBI is preferable in inpatient settings given the ability for medically supervised withdrawal and regular clinical monitoring.
• Additional research is needed to compare different buprenorphine induction methods.

Buprenorphine, 1 of 3 medications approved by the US Food and Drug Administration for opioid use disorder (OUD), is an essential treatment for addressing the widespread and highly fatal opioid epidemic.^1^^,^^2^ Its use continues to increase, including in patients using high potency synthetic opioids (HPSOs) like fentanyl, which are now widely prevalent in illicit opioid supplies.^3^^,^^4^

Buprenorphine is a partial agonist that binds to opioid receptors with high affinity but produces lower activation compared to full agonists.^5^^,^^6^ This property allows it to deter opioid use in patients with opioid dependence, as its ceiling effect prevents euphoria and overdose from full agonists.^5^ However, this same property complicates initiation. If buprenorphine is introduced while a full agonist is still present, it displaces the agonist, causing a relative decrease in receptor activation and resulting in precipitated withdrawal.^7^^,^^8^ Precipitated withdrawal, which is increasingly burdensome with widespread use of illicitly manufactured fentanyl, is often more distressing than natural withdrawal and can deter patients from future attempts to use buprenorphine as a medication for OUD (MOUD).^9^, ^10^, ^11^, ^12^, ^13^

Recent innovations in buprenorphine induction have been developed to address these challenges.^4^^,^^10^^,^^11^^,^^14^, ^15^, ^16^, ^17^ Two innovative approaches for starting buprenorphine for patients, whether already in opioid withdrawal, experiencing withdrawal after naloxone overdose reversal, or still actively using, are low- and high-dose buprenorphine induction (LDBI and HDBI, respectively).^1^^,^^16^, ^17^, ^18^, ^19^, ^20^ HDBI, defined here as 16 mg or more sublingually (SL) as an initial dose, bypasses the slow titration process used in standard inductions.^15^^,^^16^^,^^18^ This approach contrasts dosage protocols of standard inductions, with existing treatment guidelines published by the Department of Human and Health Services^21^ and found in clinical research^15^^,^^22^^,^^23^ recommending up to 16 mg during the first 24 hours of treatment. HDBI attempts to achieve therapeutic buprenorphine levels within 3-4 hours, as opposed to 1-3 days, which is typical of standard induction.^16^^,^^24^, ^25^, ^26^

LDBI is an additional innovation in the use of buprenorphine.^27^, ^28^, ^29^, ^30^ This low-dose approach is an alternative method of induction involving as little as 0.2-2 mg per day for 3-5 days, before increasing to therapeutic levels of 16-32 mg daily.^29^, ^30^, ^31^ LDBI takes advantage of slow displacement of full opioid agonist from mu-opioid receptors, thereby minimizing the risk for precipitated opioid withdrawal while also avoiding the onset of symptoms in patients not already withdrawing.^6^^,^^29^ A retrospective cohort study of 24 patients that underwent LDBI by Sokolski et al^30^ demonstrated strong initiation completion rates of 79% prior to discharge, with no patients experiencing precipitated withdrawal.

A related strategy, known as rapid LDBI or rapid micro-induction, involves the administration of small, frequent doses of buprenorphine (eg, 0.5 mg every 3 hours on day of induction with increase in dosage over 4-5-day course^23^). This strategy has also been reported as a feasible and well-tolerated alternative to traditional and slower low-dose initiations for hospitalized patients and may offer a more efficient path to stabilization while retaining the benefits of minimizing withdrawal symptoms.^11^^,^^14^^,^^23^^,^^30^

However, disadvantages of LDBI and its requirement for longer initiation include potentially longer inpatient lengths of stay relative to HBDI, particularly if efficient transitions of care are not achieved.^29^ Quicker, simpler dosing strategies for HDBI over non-rapid LDBI may also support HDBI usage.^3^ Despite successes of both HDBI and LDBI,^30^, ^31^, ^32^, ^33^ there is not sufficient evidence to suggest that either LDBI or HDBI is superior to the other.^20^

HDBI has primarily been studied in emergency department populations.^16^^,^^18^^,^^19^^,^^34^ Prior case reports have demonstrated the use of HDBI to successfully resolve buprenorphine-precipitated withdrawal, in line with its pharmacokinetics as a partial agonist.^35^, ^36^, ^37^, ^38^, ^39^ A 579-encounter case series by Herring et al^16^ found HDBI to be a safe and effective induction method with a low rate of precipitated withdrawal and no reports of oversedation. A retrospective cohort study from 2020 by CA Bridge, a California-based Public Health Institute program focused on addiction treatment and equity, showed no significant difference in follow-up engagement for patients reporting fentanyl use who received HDBI compared to those who did not report fentanyl use, with only a 1.6% incidence of precipitated withdrawal across all patients who received high-dose buprenorphine.^18^

Many patients with OUD and acute opioid intoxication are admitted to inpatient services.^19^ To further illustrate the potential safety of HDBI in inpatient hospital settings, we present theoretical cases that demonstrate this approach and its utility in patients with complex medical histories and OUD.

## Theoretical Case 1

A middle-aged female with a history of OUD, cocaine use, provoked seizures, and posterior reversible encephalopathy syndrome was admitted for seizure activity and altered mental status. She underwent metabolic and neurologic workup including electrolytes and head imaging without new acute findings, and the altered mental status was attributed to opioid use. On initial evaluation by the addiction consult service, she reported daily use of 6 bags of heroin/fentanyl, which she suspected was contaminated, possibly triggering the seizure. At the time of evaluation, she had been opioid-free for over 48 hours, with a Clinical Opiate Withdrawal Scale (COWS) score of 12, showing symptoms such as gooseflesh, abdominal cramping, diaphoresis, diarrhea, anxiety, and irritability. The patient expressed interest in starting buprenorphine, having previously had success with it during incarceration. Given her severe withdrawal symptoms and chronic fentanyl use, she was started on a high-dose buprenorphine induction of 24 mg SL, with the option for an additional 8 mg if necessary, though it was not required. High-dose induction was chosen to expedite relief from opioid withdrawal and known prior success with a total daily dose of 24 mg of buprenorphine. She reported immediate relief after the initial dose and denied any further withdrawal symptoms or adverse effects. She was continued on a maintenance dose of 24 mg daily, divided into two doses, with plans for outpatient follow-up.

## Theoretical Case 2

An elderly male with a history of OUD and asthma was admitted for acute gastrointestinal distress. While in the hospital, he intermittently received morphine and tramadol for pain management. Prior to admission, he had been using three bags of heroin daily via insufflation, though noted concern about contamination with fentanyl. He expressed a desire to stop opioid use and begin medication for OUD. He developed symptoms of opioid withdrawal 2 days after last opioid use (a dose of morphine) including nausea, abdominal cramping, diarrhea, chills, and generalized malaise. Despite these symptoms he identified the potential for still worse withdrawal symptoms. He expressed a desire to initiate buprenorphine. To avoid exacerbating his withdrawal (which was not precipitated by MOUD) and to minimize further discomfort, he was given 24 mg SL, followed by 8 mg twice daily the next day. After initiation, he reported no cravings and found buprenorphine effective in managing his withdrawal symptoms.

## Discussion

Our letter to the editor describes the theoretical use cases of HDBI for 2 patients presenting with different degrees of opioid withdrawal. HDBI may allow for rapid stabilization and reduce the need for prolonged withdrawal periods, with additional studies needed to evaluate promise in improving these outcomes and retention in care.

Per a 2024 scoping review by Wong et al,^40^ HDBI is preferable for amenable patients in inpatient settings given the ability for medically supervised withdrawal and regular clinical monitoring, especially for patients with additional comorbidities. However, no current literature exists that prospectively study whether HDBI improves the care and experience of patients with OUD relative to other initiation strategies. Additionally, HDBI is not suited for all patients, as clinicians should exercise caution with larger buprenorphine doses in patients who are more ill.^16^ While the ceiling effect of buprenorphine protects against respiratory depression,^41^ a Herring et al^16^ study of HDBI use in emergency department settings discussed the need to reexamine HDBI use in ill and intoxicated patients with reduced respiratory reserve. Additionally, patients transitioning from methadone or those with significant pain control needs requiring full opioid agonist therapy (ie, peri-operative care) may not be suitable for HDBI, and may instead be better LDBI candidates.^3^

Standard buprenorphine inductions aim to prevent precipitated withdrawal by delaying administration until the patient has abstained from opioids long enough to enter natural withdrawal.^15^^,^^22^ For heroin, this typically requires a waiting period of at least 12 hours after the last use.^5^^,^^42^ However, the growing prevalence of HPSOs such as fentanyl in illicit supplies has made standard buprenorphine inductions less effective. ^12^^,^^13^^,^^43^ In the fentanyl era, the risk of precipitated withdrawal is significantly prolonged, lasting up to 3 days, despite fentanyl being classified as a short-acting opioid.^7^^,^^42^ Patients may also present with deceptively low COWS scores due to the delayed onset of objective withdrawal signs, even amid significant psychological distress.^44^ This extended duration of withdrawal, which remains an active area of research, is often intolerable for many patients, complicating effectiveness of standard buprenorphine induction protocols.^15^^,^^44^^,^^45^ The standard induction approach often fails for patients using HPSOs in part due to protracted renal clearance (ie, fentanyl) and potential greater risk of precipitated withdrawal,^42^^,^^45^ as these patients may either be unable to tolerate withdrawal or find standard doses of buprenorphine inadequate.^3^

Predictors of precipitated withdrawal, particularly the use of HPSOs, is currently unclear and an evolving area of research.^7^, ^8^, ^9^^,^^36^ In many urban settings, where the illicit opioid supply is well-documented to predominately contain fentanyl mixed with heroin, toxicology testing beyond the standard urine drug screen, which detects morphine and its derivatives, may not be performed.^12^^,^^13^^,^^43^ A retrospective cohort study performed by Thakrar et al^7^ compared the clinical courses of 226 adults with OUD that received an initial dose of 2-4 mg of buprenorphine after COWS ≥8 versus patients that received an initial dose of ≥8 mg of buprenorphine after COWS ≥8. The study found precipitated withdrawal, which occurred in 12% of the study population, to be associated with greater fentanyl use as measured by urine concentration.^7^

A narrative review by Spadaro et al^34^ highlights how HDBI can help prevent precipitated withdrawal in patients with OUD and suggests that high-dose strategies may be more effective for patients who face longer gaps in care between discharge and outpatient follow-up. In response to promising data, many organizations, like New York’s Office of Addiction Services and Supports’ Medical Advisory Panel^19^ and CA Bridge,^46^ are now encouraging the use of HDBI for OUD management.

For some patients, outpatient buprenorphine induction may be impractical.^29^ Factors such as ongoing access to non-prescribed opioids, which increases the risk of overdose, long intervals between follow-ups, and limited ability to promptly manage withdrawal symptoms can reduce the tolerability and success of outpatient induction.^22^^,^^47^ The hospital setting offers an advantage in these cases, providing close monitoring, timely symptom management, and a controlled environment to safely achieve a therapeutic buprenorphine dose.^3^^,^^4^^,^^7^^,^^14^^,^^46^ Additionally, hospitals are better equipped to address the medical complexities often seen in patients with OUD, which may not be adequately managed in outpatient settings.^48^^,^^49^ HDBI or rapid LDBI may serve as useful tools for patients and clinicians looking to initiate inpatient treatment without delaying discharge.^17^^,^^23^^,^^30^ However, despite strong evidence supporting its effectiveness, many patients still face barriers to accessing inpatient buprenorphine treatment regardless of dosing strategy.^50^^,^^51^ These include limited provider familiarity and comfort, poor institutional support, patient misinformation, and misunderstandings about the medication’s legal status.^47^^,^^52^

More research is needed to directly compare HDBI with standard and LDBI protocols and address hospital-specific challenges to evaluate if the adoption of HDBI in hospitals may lead to better outcomes and efficiency of care for patients and providers.

## Declaration of Generative AI and AI-Assisted Technologies in the Writing Process

During the preparation of this work the authors used ChatGPT in order to improve readability and language of the manuscript. After using this tool/service, the authors reviewed and edited the content as needed and take full responsibility for the content of the publication.

## CRediT authorship contribution statement

**Scott A. Wu:** Writing – review & editing, Writing – original draft, Project administration. **Gayane N. Archer:** Writing – review & editing, Writing – original draft. **Brent D. Schnipke:** Writing – review & editing, Writing – original draft, Supervision, Project administration, Conceptualization.

## Declaration of competing interest

The authors declare that they have no known competing financial interests or personal relationships that could have appeared to influence the work reported in this paper.

## Appendix Supplementary materials

Image, application 1
