# Supplementary material for: High-dose buprenorphine inductions in hospital settings
Source: Am J Med Open. 2025 Sep 16;14:100118. doi: 10.1016/j.ajmo.2025.100118 (PMC12662114; doi:10.1016/j.ajmo.2025.100118)
Supplement: Supplementary file 1 [file mmc1.pdf]


Subject: RE: Decision on your submission to American Journal of Medicine Open
Date: Tuesday, August 5, 2025 at 12:41:56 PM Pacific Daylight Time
From: Stuart Chipkin
To: Scott Andrew Wu, AJMOpen@stellarmed.com, Megan Buresh
CC: Brent Schnipke (NM), Gayane Archer (NM), Ramondetta, Melissa (ELS-HBE)
Attachments: image001.png

Re: AJMO- AJMO-D-24-00159R3
"High dose buprenorphine inductions in hospital settings: two case reports"

Dear Mr. Wu and authors:
Thank you for your patience as we work to resolve this administrative issue regarding your manuscript.
Polices from Elsevier, which are in keeping with other publishers, require documentation of informed consent for case reports.
There are two other options which would allow the main portions of your manuscript to be published.

1. Brief Observations or Clinical Communication to the Editor: this option requires a format of: Abstract, Introduction, Methods, Results, Discussion and Conclusion.
2. Commentary or Letter to the Editor: this option is less structured and can describe an issue related to the practice of medicine.

For either of these categories, I would ask that you and your co-authors alter your manuscript and refer to "hypothetical examples" or "theoretical teaching cases" of patients who might benefit from high-dose of buprenorphine inductions. It would be best to keep all references very generic; for example instead of a specific age refer to a decade or other qualifier. The emphasis is to have your manuscript change from a report of two cases to a discussion of your proposed approach.

For an example of this approach, please see a recently published article:
Cardiovascular-Kidney-Metabolic (CKM) Syndrome: A Case-Based Narrative Review - ScienceDirect

If you and your authors are willing to adopt these suggestions, your new version will be reviewed quickly by the relevant editorial board members and we will provide you with an expedited decision.

Again, my apologies for the way this has unfolded but we are under an obligation to abide by standard rules for case reports and informed consent.

With regards,

Stuart Chipkin, MD
Editor-in-chief, American Journal of Medicine Open

Megan Buresh, MD
Associate Editor, American Journal of Medicine Open
Guest Editor, Virtual Special Issue on Care of Hospitalized Patient with Substance Use Disorders

---

From: Scott Andrew Wu <scott.wu@northwestern.edu>
Sent: Monday, July 28, 2025 3:15 PM
To: Stuart Chipkin <schipkin@kin.umass.edu>; AJMOpen@stellarmed.com; Megan Buresh <mburesh2@jhmi.edu>
Cc: Brent Schnipke (NM) <brent.schnipke@nm.org>; Gayane Archer (NM) <gayane.archer@nm.org>; Ramondetta, Melissa (ELS-HBE) <m.ramondetta@elsevier.com>
Subject: Re: Decision on your submission to American Journal of Medicine Open

You don't often get email from scott.wu@northwestern.edu. Learn why this is important

Dr. Chipkin,

Thank you very much for your note and no worries at all.

Please let us know how this goes and how we might be able to tailor our manuscript for publication given ongoing conversations with Dr. Buresh and the publisher team. We are glad the educational value of the submission is apparent and hope to see it published.

Thank you again,
Scott & Author Team

From: Stuart Chipkin <schipkin@kin.umass.edu>
Date: Monday, July 28, 2025 at 11:31 AM
To: Scott Andrew Wu <scott.wu@northwestern.edu>, AJMOpen@stellarmed.com <AJMOpen@stellarmed.com>, Megan Buresh <mburesh2@jhmi.edu>
Cc: Brent Schnipke (NM) <brent.schnipke@nm.org>, Gayane Archer (NM) <gayane.archer@nm.org>, Ramondetta, Melissa (ELS-HBE) <m.ramondetta@elsevier.com>
Subject: RE: Decision on your submission to American Journal of Medicine Open

Dear Dr. Wu:
My apologies for the delay in getting back to you.
This situation has been a bit challenging. I've been speaking with Dr. Buresh and the publisher about this situation.
The unfortunate reality is that your submission as a case report should not have been considered without documentation of informed consent.
However, we recognize that you should have been notified much sooner in the process. In addition, Dr. Buresh and I feel there is merit to the educational message of your submission.

We're trying to see if we can come up with an option that will meet the necessary regulatory requirements but also allow you publish your important manuscript in American Journal of

Medicine Open.

I'd like to ask for your patience a bit longer while we work to come up with a successful resolution.

Thank you for your understanding.

Stuart R. Chipkin, MD
Research Professor
School of Public Health and Health Sciences
University of Massachusetts Amherst
Amherst, MA 01003-9304

Editor-in-Chief
American Journal of Medicine Open
@AjmOpen

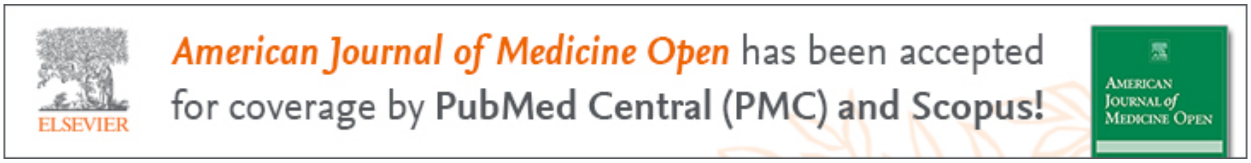

From: Scott Andrew Wu <scott.wu@northwestern.edu>
Sent: Wednesday, June 25, 2025 10:37 PM
To: Stuart Chipkin <schipkin@kin.umass.edu>; em.ajmo.3e7.940df9.06744691@editorialmanager.com; AJMOpen@stellarmed.com
Cc: Brent Schnipke (NM) <brent.schnipke@nm.org>; Gayane Archer (NM) <gayane.archer@nm.org>
Subject: Re: Decision on your submission to American Journal of Medicine Open

You don't often get email from scott.wu@northwestern.edu. Learn why this is important

Dr. Chipkin and AJMO Editorial Team,

I hope you are doing well. I wanted to reach out again to check if our question about the manuscript cases and consent process had been received.

I've included a couple extra email addresses that may be relevant. We appreciate your time and consideration.

Thank you,
Scott

Scott Wu
Northwestern University
Cell: 925-818-0210

From: Scott Andrew Wu <scott.wu@northwestern.edu>
Date: Tuesday, June 17, 2025 at 7:13 AM
To: Stuart R. Chipkin <schipkin@kin.umass.edu>
Cc: Brent Schnipke (NM) <brent.schnipke@nm.org>, Gayane Archer (NM) <gayane.archer@nm.org>
Subject: Re: Decision on your submission to American Journal of Medicine Open

Dr. Chipkin & AJMO Editorial Team,

Thank you for your note and continued support of our manuscript.

Our author team has discussed the written consent issue and attempted to reach out to the patients included in the two case reports, but are unable to obtain these consents. Given these issues, we are asking if this requirement can be waived for this submission, or if we can trim the case reports or make them more composite.

Originally, this manuscript took shape as a research letter or advocacy piece with literature review. Perhaps if we reverted to this structure with the core parts of the manuscript that have already been deemed acceptable, this may be a viable solution.

We look forward to any feedback on this matter. Thank you again for your time.

Best,
Scott

Scott Wu
Northwestern University
MD-MBA Student | Class of 2025
Cell: 925-818-0210

From: em.ajmo.3e7.940df9.06744691@editorialmanager.com <em.ajmo.3e7.940df9.06744691@editorialmanager.com> on behalf of Stuart R. Chipkin <em@editorialmanager.com>
Date: Friday, June 13, 2025 at 9:36 AM
To: Scott Andrew Wu <scott.wu@northwestern.edu>
Subject: Decision on your submission to American Journal of Medicine Open

Manuscript Number: **AJMO-D-24-00159R3**
High dose buprenorphine inductions in hospital settings: two case reports

Dear Mr. Wu,

Thank you for submitting your manuscript to American Journal of Medicine Open. I am sorry for the confusion with the previous acceptance.

I have been in contact with the journal's publisher, and it is necessary to point out that we cannot accept a case report for publication without confirming the patient has provided cosent for the details to be published. I am sorry this was not discovered earliner. See below:

Informed consent and patient details

Key guidelines:

- Appropriate consents, permissions and releases must be obtained if case details, personal information and images of patients or any other individuals are included in a publication, even if anonymized.
- Patient and research subjects' names, initials, hospital or social security numbers, dates of birth or any other personal or identifying information should never be used, even where consent has been provided.

Written consents must be retained.

Please resubmit the case for final approval after appropriate consents have been obtained.

To submit your revised manuscript, please log in as an author at https://www.editorialmanager.com/ajmo/, and navigate to the "Submissions Needing Revision" folder under the Author Main Menu.

Research Elements (optional)
This journal encourages you to share research objects - including your raw data, methods, protocols, software, hardware and more – which support your original research article in a Research Elements journal. Research Elements are open access, multidisciplinary, peer-reviewed journals which make the objects associated with your research more discoverable, trustworthy and promote replicability and reproducibility. As open access journals, there may be an Article Publishing Charge if your paper is accepted for publication. Find out more about the Research Elements journals at https://www.elsevier.com/authors/tools-and-resources/research-elements-journals?dgcid=ec_em_research_elements_email.

American Journal of Medicine Open values your contribution and I look forward to receiving your revised manuscript.

Kind regards,

Stuart R. Chipkin, MD
Editor-in-Chief
American Journal of Medicine Open

More information and support

FAQ: How do I revise my submission in Editorial Manager?

https://service.elsevier.com/app/answers/detail/a_id/28463/supporthub/publishing/
FAQ: How can I reset a forgotten password?
https://service.elsevier.com/app/answers/detail/a_id/28452/supporthub/publishing/
For further assistance, please visit our customer service site:
https://service.elsevier.com/app/home/supporthub/publishing/
Here you can search for solutions on a range of topics, find answers to frequently asked questions, and learn more about Editorial Manager via interactive tutorials. You can also talk 24/7 to our customer support team by phone and 24/7 by live chat and email

At Elsevier, we want to help all our authors to stay safe when publishing. Please be aware of fraudulent messages requesting money in return for the publication of your paper. If you are publishing open access with Elsevier, bear in mind that we will never request payment before the paper has been accepted. We have prepared some guidelines (https://www.elsevier.com/connect/authors-update/seven-top-tips-on-stopping-apc-scams) that you may find helpful, including a short video on Identifying fake acceptance letters (https://www.youtube.com/watch?v=o5l8thD9XtE). Please remember that you can contact Elsevier s Researcher Support team (https://service.elsevier.com/app/home/supporthub/publishing/) at any time if you have questions about your manuscript, and you can log into Editorial Manager to check the status of your manuscript (https://service.elsevier.com/app/answers/detail/a_id/29155/c/10530/supporthub/publishing/kw/status/).

In compliance with data protection regulations, you may request that we remove your personal registration details at any time. (Remove my information/details). Please contact the publication office if you have any questions.
